# Exploring the utility and scalability of a telehomecare intervention for patients with chronic kidney disease undergoing peritoneal dialysis—a study protocol

**DOI:** 10.1186/s12882-017-0557-y

**Published:** 2017-05-10

**Authors:** Lianne Jeffs, Arsh Kumar Jain, Rachel HiuTung Man, Nike Onabajo, Laura Desveaux, James Shaw, Jennifer Hensel, Payal Agarwal, Marianne Saragosa, Trevor Jamieson, Ivy Wong, Maria Maione, R. Sacha Bhatia

**Affiliations:** 1 Keenan Research Centre, Li Ka Shing Knowledge Institute St Michaels Hospital, 209 Victoria St, Toronto, ON M5B 1T8 Canada; 2 London Health Sciences Centre, 800 Commissioners Rd E, London, ON N6A 5W9 Canada; 3 Women’s College Hospital, 76 Grenville St, Toronto, ON M5S 1B2 Canada

**Keywords:** Chronic kidney disease, Telehomecare, Realist evaluation

## Abstract

**Background:**

Chronic Kidney Disease (CKD) is a pressing global health concern that is placing increased strain on health care resources. CKD patients regularly receive peritoneal dialysis as a common CKD treatment. An emerging technological solution is telehomecare as way to support patients receiving PD in their homes. This study protocol outlines a mixed methods evaluation exploring a telehomecare developed to enhance CKD patients’ outcomes and experiences. The study aims to assess the usability, acceptability and scalability of this virtual care application.

**Methods:**

A realist evaluation using an embedded case study design will be used to understand the usability, acceptability and scalability of a telehomecare application for patients with CKD undergoing PD. The realist evaluation that is further described in this paper is part of a larger evaluation of the eQ Connect™ intervention that includes a randomized, parallel-arm control trial aimed at determining if utilizing eQ Connect improves selected clinical outcomes for PD patients (CONNECT Trial).

**Discussion:**

Potential implications of this study include elucidating which components of the intervention are most effective and under what conditions with a focus on the contextual influences. Collectively, our multi-method design will yield knowledge around how best to implement, sustain and spread the telehomecare application that will be useful to guide the development, implementation and evaluation of future virtual care applications aimed at improving the quality of care outcomes and experiences of patients.

**Trial registration:**

NCT02670512. Registered: January 18, 2016.

## Background

Over 70 million individuals worldwide have Chronic Kidney Disease (CKD) making it a pressing global health concern that is placing increased strain on health care resources related to CKD care [1, 2]. The prevalence of CKD stages 1 to 4 has increased dramatically from 10% in 1988–1994 to 13% in 1999–2004 in the United States [3] and 12.5% (representing 3 million adults) from 2007 to 2009 in Canada [4]. These higher rates of chronic disease may also lead to adverse outcomes and end stage renal disease (ESRD) requiring dialysis or kidney transplantation [5]. Globally, approximately 190,000 patients regularly received peritoneal dialysis (PD) [6] which is usually performed at home daily by the patient after receiving education and training by a dialysis health care professional [7, 8]. PD has been associated with a survival advantage, especially in the first few years of therapy [9–11]; is the least costly of all the forms of dialysis (e.g. in Canada it has been recently estimated that maintaining a patient on PD compared to hemodialysis saves the healthcare system over $150,000 over a three year period [12]; and higher levels of satisfaction with care than patients receiving hemodialysis [7]; and higher health-related quality of life (HrQOL) scores [13, 14].

Regardless of treatment for CKD, care is complex and requires significant personal involvement to integrate medication adherence, lifestyle modification and nutritional adaptation into a daily routine [15]. CKD patients are often not satisfied with their communication with their health care providers and are frequently unaware of their diagnosis or its implications [16–18]. This may be due in part to the volume of CKD patients which have resulted in nephrologists seeing more patients in less time [19]. Simultaneously, alternative methods of communication are being developed and embraced by a population with growing computer literacy [20]. Emerging new technological solutions including the variety of applications and portals for health information are offering patients a better understanding of their diseases and evidence informed practice [19].

One emerging technological solution is telehomecare, which refers to a model of care using information, communications, measurement and monitoring technologies to enable healthcare providers to link with patients at home [9, 21]. Telehomecare has been associated with reduced risk of disease and other health-related problems and improved recovery in other patient populations [22, 23]. Specific examples include less symptoms and fewer hospitalizations for persons with heart failure [22], increased compliance rates and feedback in cardiac rehabilitation patients [23], and improved quality of life indicators in the pediatric palliative care population [24]. Further, telehomecare can reduce costs associated with improved coordination, continuity of care, and access to specialized care [20, 25, 26].

Although promising signs are emerging with various technologically enabled virtual care solutions for a variety of chronic illnesses, few are being employed with CKD [19]. Given that PD is performed in a home setting, patients using this modality are ideal candidates for support and monitoring using telehomecare services. Successful implementation of telehomecare is a complex, multi-factorial endeavor [27]. Although there is growing support for more process evaluation [28], much less attention is being paid to the mechanisms for delivering interventions [29] and the future scalability of interventions [27, 28, 30]. In this context, this paper provides the protocol for a realist evaluation that aims to understand how a telehomecare application aimed at enhancing CKD patients’ outcomes and experiences is implemented in practice, and to assess the usability, acceptability and scalability of this virtual care application. Realist evaluations are used to identify which components of the intervention are most effective and under what conditions with a focus on the contextual influences [29, 31, 32].

## Methods

### Study design

A realist evaluation [29, 31, 32] using an embedded case study design [33] will be used to understand the usability, acceptability and scalability of a telehomecare application for patients with CKD undergoing PD. Through this type of design, researchers are able to understand how and why the implementation succeeds or fails. A realist evaluation 1) provides an explanation for why study outcomes occur; 2) involves multi-methods involving quantitative and qualitative approaches; and 3) uses a theory-driven approach that guides the study design [32].

This realist evaluation will be guided by the RE-AIM Conceptual Framework [34] and the Institute of Healthcare Improvement (IHI)’s Triple Aim [35] and Framework for Going to Full Scale [36]. These were selected by the research team to provide an evidence-informed, comprehensive, and contemporary framework to guide the study. Key concepts of the RE-AIM framework that underpin scalability include reach, effectiveness, and adoption (see Table 1). IHI’s Triple Aim focuses on the extent to which health care innovations result in 1) improved population health, 2) enhanced patient experience, and 3) reduced health care costs, synergistic with the RE-AIM model’s effectiveness domain [35]. The phases of the IHI framework include 1) set-up, 2) develop the scalable unit, 3) test of scale-up, and 4) go to full scale [36]. Key elements include adoption mechanisms (i.e., leadership engagement, communication methods, leveraging social networks, and building a culture of urgency and persistence); support systems needed to achieve large-scale programming (i.e., a learning system that connects adopters and experts, a data system to support measurement for improvement, infrastructure such as IT, equipment, etc.), building capability through training and support, and building reliable processes that support sustainability [36].

**Table 1:** RE-AIM Framework

RE-AIM Framework Dimension	Description
Reach	Our scalability assessment will pay close attention to the “reach” of the telehomecare initiatives, which has important implications for their potential implementation across the province.
Effectiveness	We will explore perceptions and experiences associated with their participation in the demonstration initiative guided by the Institute for Healthcare Improvement’s (IHI) Triple Aim of 1) improving population health, 2) enhancing patient experience, and 3) reducing health system costs.
Adoption	Our process evaluation will explore the extent to which health care practitioners and other key stakeholders agree to deliver the intervention as intended.
Implementation	Our process evaluation will focus explicitly on how and why the telehomecare initiatives are implemented in particular ways across different contexts.
Maintenance	Our scalability assessment includes the assessment of whether key stakeholders would continue delivering the telehomecare initiatives outside the scope of the research study over time.

For this study, we hypothesize that successful adoption and scalability of the intervention will be influenced by a series of mechanisms including leadership engagement and culture, communication methods, social network, and reliable processes; structures including a learning system that includes training and support and infrastructure that connects adopters and experts, a data system to support measurement for improvement; that result in positive patient related outcomes and experiences.

### Intervention description

**Fig. 1:**
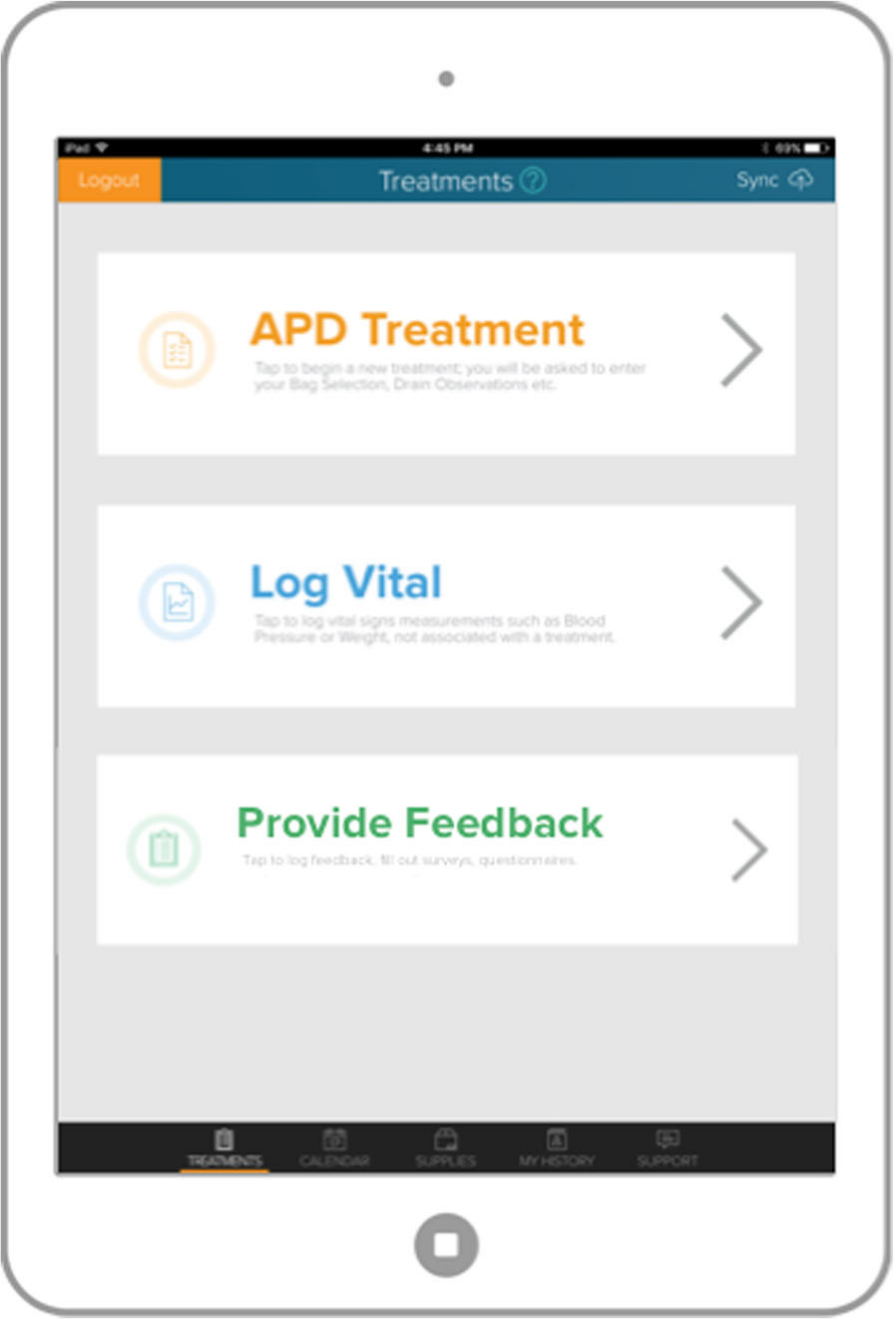
eQConnect Support Portal

The CONNECT Trial Research Program was established to further investigate a telehomecare monitoring software (eQ Connect™ system, developed by eQOL Inc.) intended for use by patients on PD. The software provides support for patients undergoing PD through enabling communication between patients and the healthcare team, providing up-to-date patient health information, and ensuring patient compliance with PD regimens, and is intended to ultimately reduce barriers for PD uptake. The patient-facing part of eQ Connect™ (Patient Portal) is built to run on a mobile tablet, allowing for mobility, uncomplicated data recording, and ease of uploading data securely over the Internet. Information such as treatment progress, health status, supply usage, etc. is transferred to a secure data center when an Internet connection is available and the information is accessible to the patient’s health care team from this center. The clinician-facing part of eQ Connect™ (Support Portal) is built on web technologies. See Fig. 1. Clinicians are able to gain access to the information entered by the patient through the application after securely logging in from any Internet web browser. This reduces the need to install additional software thereby utilizing existing computer infrastructure within hospitals and clinics.

The realist evaluation that is further described in this paper is part of a larger evaluation of the eQ Connect™ intervention that includes a randomized, parallel-arm control trial (RCT) aimed at determining if utilizing eQ Connect improves selected clinical outcomes for PD patients (CONNECT Trial). The trial expects to enroll 500 APD/CAPD patients (250 intervention, 250 control) from across Canada.

One-year data was obtained from PD patients at London Health Sciences Centre to generate the baseline estimates of rates of technique failure, infections and hospitalizations. To determine the average rate of the outcome, we calculated the proportion of patients having at least one event, and cumulative incidence of the first and all events. The rate per 1-person time year can be calculated as a crude total, a crude average or from the regression model for count data. This resulted in rates ranging from 0.8766 to 3.4. Four different sample sizes equations were used to calculate the desired sample size based on our proposed analytic plan, which includes: proportions (generally considered conservative), based on the Cox PH/Poisson regression, inflating the Cox regression for correlation within patients and based on Lachin's formula (allowing for time and incompletely follow up). All sample size equations were calculated using a 5% two-sided level of significance, 80% power and assuming a 1 year follow up. Based on the baseline estimates, we allowed parameter values to vary as follows: baseline rate per patient-year from 0.8 to 3.4 and relative risk reduction of 0.2 to 0.35 (by 0.05). Given the funding sources and availability of patients, we can realistically enroll approximately 500 patients. Using the Lachin formula, we are adequately powered to detect a 0.25 or higher relative risk reduction for baseline rates of 2.0 or higher. This is based on a sample size equation that allows for rates with variable follow up time per patient.

Recruitment will occur continuously over a 2 year period to meet the target participant population. The study protocol for this trial has been approved by the Western Research Ethics Board (#107839) through Clinical Trials Ontario and recruitment is in progress.

#### Setting

Participants will be recruited from two hospitals, one urban teaching hospital (London Health Sciences Centre) and one community hospital (Humber River Hospital), both located in Ontario Combined, there are over 200 PD patients receiving care from the two centres. Eligible patients will be approached during their regularly scheduled clinic visit at the PD clinic.

### Objectives

The objectives of the qualitative realist evaluation component of this study is to 1) understand how the eQ Connect™ application is implemented in practice, (2) explore participants’ perspectives of the usability and acceptability of the eQ Connect™application, and 3) examine the key issues associated with scaling applications such as eQ Connect™.

### Quantitative component - outcome measures

Data regarding Patient Reported Outcomes and Experiences (PROMs/PREMs) will be collected by administering a short survey to patients enrolled in the trial by providing an information letter. These standardized PROMs/PREMs will be collected at baseline and 3 months for about 200 participants in both study arms. Patients will be consented into this study at the same time they are consented into the clinical trial study. Baseline data will be collected in-person at the clinic during the RCT baseline data collection. The 3 months data collection which will include the administration of a Mobile App Rating Scale (adapted version) [37] will be synchronized with the 6 month follow-up interview for the RCT. The questionnaires – Patient Reported Costs and Patient Reported Experience - are based on the Better Access and Care for Complex Needs (BeACCoN) Measurement Framework for Evaluation of Primary and Integrated Health Care Innovation for Individuals with Complex Needs. This framework is adapted from the Commonwealth Fund Survey, Patients with Complex Needs and the Canadian Institutes of Health Research Community Based Primary Health Care Survey [38].

### Qualitative component

The qualitative component will include interviews with key stakeholders involved in the implementation process. Specifically, interviews will take place either face-to-face or in a private space at the hospital or through a telephone conversation. Interviews will be conducted between baseline and 3 months. Participants recruited for interviews will include 10 to 12 patients (caregivers will be invited to participate in those interviews as well); 4 to 6 health care providers involved in the implementation process (including at least one physician in each location); 2–4 organizational leaders who oversee the implementation process (for example, Clinic Managers); and 5–7 health system decision makers involved in the implementation of virtual care initiatives in Ontario.

Qualitative interviews will include questions about 1) participants’ experiences of learning about and using the technology; 2) changes to health care provider workflow required to effectively use the technology; 3) organizational changes required to support the technology; and 4) health system barriers and facilitators to effective implementation and evaluation. The interview guide also includes specific questions related to the feasibility of the evaluations taking place, offering participants the opportunity to provide direct input into the implementation and rapid evaluation framework. These qualitative interviews will be analyzed using thematic analysis strategies [39] by the investigative team to identify key themes related to the implementation an evaluation of the virtual care initiatives in actual contexts of health care delivery in Ontario.

### Recruitment

#### Patients/care-givers

Potential participants will be identified prospectively by a research coordinator during the RCT process. Interested patients will then be consented for the quantitative phase as per the materials and processes outlined in the quantitative protocol. Baseline data will also be collected at this time as per the quantitative protocol. During this initial consent process, an Information Letter outlining the PROMs and PREMs and the qualitative phase of the study will be distributed to individuals who consent to participation in the overall trial. The qualitative component is optional and participants who express interest, the research coordinator will obtained the potential study participants name and contact information which will then be provided to the qualitative researcher coordinator. The qualitative research coordinator will then subsequently contact the potential study participant and provide an overview of the study; obtain consent for interested study participants; schedule and conduct the interview.

#### Health care providers/administrators/stakeholders

Qualitative research team will be introduced to the HCPs at the study sites to introduce the qualitative evaluation. The qualitative component for the HCPs consists of an individual interview (either in person or via telephone).

#### Organization leaders

The Organization Leaders (OLs) names will be forwarded to the qualitative team members by the implementation team and the clinical site leads. Potential participants will be notified that they will be contacted via email by the qualitative research team. Further potential OL participants will be identified by asking each participant for further suggestions regarding individuals to contact for potential participation.

#### Health system decision makers

As a first wave of recruitment, the research team will ask the project evaluation contact to identify key individuals involved in the selection and procurement of virtual care technologies, and their subsequent introduction into the health care system. Potential participants will be notified that they will be contacted via email by the qualitative research team and an email invitation will be sent to individuals by the research coordinator.

As a second wave of recruitment, we will use snowball sampling by asking interview participants to identify other health system decision makers who are involved in the processes of around the selection, procurement, implementation, and ongoing support of telehomecare technologies.

### Data analysis

#### Quantitative data

PROMs and PREMs will be analyzed utilizing an intention to treat principle. Patient characteristics will be summarized as means and 95% confidence intervals, medians and interquartile ranges, and frequencies. Differences in patient characteristics across study arms will be compared using independent sample t-tests, Wilcoxon-Mann-Whitney tests, and/or Chi-square tests, as appropriate. The mean difference will be used to compare PROMs and PREMs between the intervention and control arms at 6 months. Within-group outcomes will be analyzed using paired t-tests and Wilcoxon-Mann-Whitney tests. All statistical analyses will be performed using SAS version 9.4 (SAS Institute, Cary, NC).

#### Qualitative data

Written observations and qualitative interviews will be immediately transcribed into word documents and prepared for qualitative analysis and analyzed using thematic analysis [39]. Specifically, analyses will be conducted with a coding schema to be constructed and used to categorize the narrative text. This analytical process involves the researchers reviewing the transcripts line-by-line separately to identify sections of text that serve as codes; the researchers will meet to determine the codes and categories through consensus; and the final step the researchers develop themes from the categorical data through consensus.

The quantitative and qualitative datasets will be triangulated and compared and contrasted to the RE-AIM and IHI frameworks. Methods to ensure conceptual and methodological rigor will be employed to ensure assure confirmability, dependability, and credibility of the data interpretation.^34^ Using a series of analytical sessions with the research team and knowledge users, a case report will be finalized. This analysis will also yield a series of iteratively refined statements of the relationships between 1) key contextual factors, 2) the mechanisms by which they effect the implementation of the virtual care interventions, and 3) the impact on the outcomes of the intervention themselves referred to as “Context-Mechanism-Outcome Configurations” [29, 31, 32]. These statements will then be used to revise the framework to more accurately reflect the key contextual influences and practices that constitute the implementation process of virtual applications (in this context telehomecare) to be used to “scale-up” the intervention across health care settings in Ontario.

### Strengths and limitations

There are limitations inherent in our design including the generalizability of the quantitative data and the transferability of the qualitative data to other types of healthcare organizations due to study being conducted at two hospitals at a single site large acute care teaching hospital. There are also selection biases that are inherent in study participants volunteering to participate.

With our methodological approach, we aim to enrich the evolving field of virtual care applications (in our case telehomecare intervention) and implementation science. Our approach includes multiple sources of data aimed at elucidating the interpretations and actions of a diverse group of stakeholders triangulated with patient outcomes and experience measures. This includes gaining insights into how patients respond to the idea of the telehomecare interventions (whether certain populations were interested or not) and how they react to the interventions themselves (whether they viewed the experience positively). Our prospective realist evaluation study design approach will also capture the utility, benefits, and unintended consequences and our scalability assessment will inform future efforts to how best to ‘scale up’ virtual care applications. Collectively, this study has the potential to contribute to accelerating knowledge by producing a comprehensive dataset on how remote telemonitoring can improve health care outcomes of peritoneal dialysis patients.

## Discussion

Our study design will provide insight into how a telehomecare application aimed at enhancing CKD patients undergoing PD outcomes and experiences is implemented in practice, and the usability, acceptability and scalability of this virtual care application. In addition to examining patient outcomes and experiences, we will elucidate which components of the intervention are most effective and under what conditions with a focus on the contextual influences. Collectively, our multi-method design will yield knowledge around how best to implement, sustain and spread the telehomecare application that will be useful to guide the development, implementation and evaluation of future virtual care applications aimed at improving the quality of care outcomes and experiences of patients.

### Study status

The study is actively recruiting participants from one of the two sites. One participant has been recruited to date.

## Abbreviations

BeACCoN: Better access and care for complex needs
CKD: Chronic kidney disease
ESRD: End Stage renal disease
HCP: Health care professionals
HrQOL: Higher health-related quality of life
IHI: Institute of healthcare improvement
OL: Organization leader
PD: Peritoneal dialysis
PROMS/PREMS: Patient reported outcomes and experiences
RCT: Randomized, parallel-arm control trial
